# A Wirelessly Controlled Scalable 3D-Printed Microsystem for Drug Delivery

**DOI:** 10.3390/ph14060538

**Published:** 2021-06-04

**Authors:** Farzad Forouzandeh, Nuzhet N. Ahamed, Xiaoxia Zhu, Parveen Bazard, Krittika Goyal, Joseph P. Walton, Robert D. Frisina, David A. Borkholder

**Affiliations:** 1 Department of Microsystems Engineering, Rochester Institute of Technology, Rochester, NY 14623, USA; ff7667@rit.edu (F.F.); nn5878@rit.edu (N.N.A.); kxg5867@rit.edu (K.G.); 2 Department of Medical Engineering, Global Center for Hearing & Speech Research, University of South Florida, Tampa, FL 33620, USA; xiaoxiazhu@usf.edu (X.Z.); parveen1@mail.usf.edu (P.B.); jwalton1@usf.edu (J.P.W.); rfrisina@usf.edu (R.D.F.); 3 Department of Chemical, Biological & Materials Engineering, University of South Florida, Tampa, FL 33620, USA; 4 Department of Communication Sciences & Disorders, Global Center for Hearing & Speech Research, University of South Florida, Tampa, FL 33620, USA

**Keywords:** drug delivery, micropump, microreservoir, 3D printing, implantable, transdermal

## Abstract

Here we present a 3D-printed, wirelessly controlled microsystem for drug delivery, comprising a refillable microreservoir and a phase-change peristaltic micropump. The micropump structure was inkjet-printed on the back of a printed circuit board around a catheter microtubing. The enclosure of the microsystem was fabricated using stereolithography 3D printing, with an embedded microreservoir structure and integrated micropump. In one configuration, the microsystem was optimized for murine inner ear drug delivery with an overall size of 19 × 13 × 3 mm^3^. Benchtop results confirmed the performance of the device for reliable drug delivery. The suitability of the device for long-term subcutaneous implantation was confirmed with favorable results of implantation of a microsystem in a mouse for six months. The drug delivery was evaluated in vivo by implanting four different microsystems in four mice, while the outlet microtubing was implanted into the round window membrane niche for infusion of a known ototoxic compound (sodium salicylate) at 50 nL/min for 20 min. Real-time shifts in distortion product otoacoustic emission thresholds and amplitudes were measured during the infusion, demonstrating similar results with syringe pump infusion. Although demonstrated for one application, this low-cost design and fabrication methodology is scalable for use in larger animals and humans for different clinical applications/delivery sites.

## 1. Introduction

Over the past few decades, controlled drug-delivery systems have drawn researchers’ attention due to enhanced therapeutic interventions by enabling consistent drug delivery over time and improving patient comfort, safety, and compliance. Microscale reservoir-based systems have been employed in consistent administration and site-directed delivery to relieve systemic exposure to the drug and chemical side effects [1]. The miniaturized size of such systems assists local delivery to relatively inaccessible sites or specific tissues in small rodents and humans. Additionally, the pumping mechanisms in these devices enable precise delivery of biotherapeutics in ultra-low infusion ranges of 0.01–1 µL/min, minimizing mechanical damage in the target tissues.

Reservoir-based drug-delivery devices with volumes of a few hundred microliters have widespread applications in implantable and transdermal drug delivery. Implantable drug-delivery systems address current medical needs by enabling elegant and innovative drug-delivery concepts [1]. These devices usually store the drug in a reservoir while a micropump is used to pump the drug from the reservoir to the target organ via catheter microtubing. Implantable drug-delivery systems with reservoir capacities of 1.2, 15, 20, 60, and 130 µL were used for drug delivery to the posterior segment of the eye [2], the rapid vasopressin release for treating hemorrhagic shock and ambulatory emergency care [3,4], the delivery of ranibizumab into the vitreous cavity [5], and guinea pig inner ear drug delivery [6], respectively. Transdermal drug-delivery devices have largely been used because they offer unique features such as low sustainable cost, minimal invasiveness, ease of use, and capability of self-administration [7]. These devices generally consist of a reservoir, a microneedle mechanism, and a pumping mechanism for efficient delivery through the microneedles [8,9]. Transdermal drug-delivery devices with reservoir capacities of 5, 12, 20, 70, and 100 µL were used for trace blood tests [10], insulin administration to diabetic rats [11], general pharmaceutical applications [12], fast-onset and sustained delivery of lidocaine [13], and general transdermal drug-delivery applications [8], respectively.

Micropumps provide the driving force to propel the drugs in active drug-delivery microsystems and have been used widely in reservoir-based drug-delivery microsystems. Among various micropumps concepts, peristaltic micropumps are one of the most suitable ones for drug-delivery applications because they are simple to miniaturize and fabricate on a single substrate, reliable, enduring, tolerant to bubbles, and capable of self-priming and handling viscous working fluids [14]. In these micropumps, typically two or more chambers are actuated sequentially to create pumping and rectification for the working fluid. The peristalsis pumping concept enables the realization of a micropump by building multiple actuating chambers around a single microtubing, which makes the structure readily scalable and leak-free by eliminating interconnection in the system, which is critical in ultra-small microtubing [15]. Micropumps for drug-delivery applications must be small, thin, and self-contained, preferably with the control electronics integrated with the system. In addition, for most applications, these micropumps should be programmable and wirelessly controlled, for simple access by the user/care provider to the delivery profile, timing, and frequency during the day.

Most of the drug reservoirs in drug-delivery systems are designed to be refillable using refill ports to reduce the overall size of the system and eliminate the need for a large reservoir [7]. Refill ports utilize a baseplate for penetration-depth limitation and a cavity between the septum and baseplate with an exit channel to provide a fluidic interconnection to a storage area [16,17,18]. To assist palpation, localization, or visual identification for users, some systems incorporate a raised ridge into the refill port [18,19]. Sharp, thin, non-coring needles are used for refilling the reservoir by injecting into a septum located in a designated refill port in the system housing [20,21]. The septa are generally made of silicone rubber [20] due to its high resilience [22], high deformability, self-adherence [23,24], and self-healing capabilities [25]. The numbers of leakage-free punctures reported in the literature or on the market are between 100 and 1000 with more than 3 mm septum thickness [19,26], and less than 100 with submillimeter septum thickness [27,28,29,30]. Another important feature of non-pressurized reservoirs is that ideally, they should act as a storage unit with no impact on pumping performance. However, most reservoirs are made of elastomeric membranes integrated into pumping mechanisms that deform due to the extraction of fluid due to pumping, resulting in a restoring force, which tends to restore the membrane to its original shape. After pumping has stopped, the restoring force results in a non-negligible backward flow, which is typically counteracted by employing either a normally closed fluidic channel or check valves [27,31,32]. This restoring force prevents the reservoir from being integrated into normally open pumps due to its dramatic effect on the pumping efficiency.

Laboratory animal models have proven to play key roles in understanding and treating human diseases. Rats and mice are two of the most prevalent animal models in research [33], with mice being capable of being genetically manipulated [34]. Proof-of-concept development in smaller animal models requires miniaturization due to the size limitation and scalability of the design, and fabrication to enable clinical translation. The minimization of the overall thickness of the implantable system is another essential feature for animal model drug-delivery systems and in size-constrained transdermal and implantable drug-delivery systems in humans. 3D-printing technology has emerged as a powerful tool in the creation of miniaturized drug-delivery systems by enabling the fabrication of thin, miniature, and scalable devices with biocompatible materials. 3D printing is preferred to typical fabrication methods of microsystems (e.g., microelectromechanical systems and conventional machining) due to inexpensive and quick prototyping, inexpensive fabrication facility maintenance, sufficient miniaturization, scalability, and biocompatibility.

Here, we present a low-cost, miniature, thin, programmable, and wirelessly controlled microsystem for implantable and transdermal drug delivery, fabricated with 3D-printing technology and enabling scalability for applications ranging from small animal models to clinical use in children and adults. We review the designs of the microreservoir and the micropump, which were published recently [35,36], along with describing modifications of the micropump fabrication process to improve long-term application suitability. The micropump and microreservoir were integrated in a single embodiment using 3D-printing technology. In one configuration, the microsystem was optimized for implantable inner-ear drug delivery for murine models. This application necessitated miniaturization and a thin form factor of the device for subcutaneous implantation in one of the smallest animal models, and ultra-low flow rates to minimize damage to the target organ, the mouse cochlea, with an overall fluid volume of only 620 nL [37]. For this purpose, a 10 µL reservoir was integrated with the micropump with a catheter microtubing of 125 µm ID and 250 µm OD, providing flow rates in the range of 10–100 nL/min. The suitability of the device for in vivo drug delivery was tested by benchtop experiments to measure long-term flow-rate stability and accuracy, and by exploring long-term biocompatibility. In vivo results indicated functional round window membrane (RWM) drug delivery, consistent with the results of benchtop syringe pumps.

The micropump structure was built directly on the back of a printed circuit board assembly integrated with the mechanical and control electronic components, and around a catheter microtubing to provide a leak-free flow path. The micropump operates on the peristalsis concept by having three chambers that surround the microtubing, actuated by expansion/shrinkage of a thermal phase-change material (paraffin wax) due to its melting/crystallization. The micropump is readily scalable by modification of the microtubing and chamber size [36]. The microreservoir structure was built using 3D-printing technology, with a thin dome-shaped parylene-C membrane for a restoring force-free drug storage, and an ultra-thin silicone-based septum. The entire interior surface of the microreservoir is coated with parylene-C, providing a class VI biocompatible environment for long-term drug storage. 3D-printing technology enables scalability in the range of 1–100 µL and integration to alternative pumping mechanisms by minimal change in the design [35].

**Figure 1:**
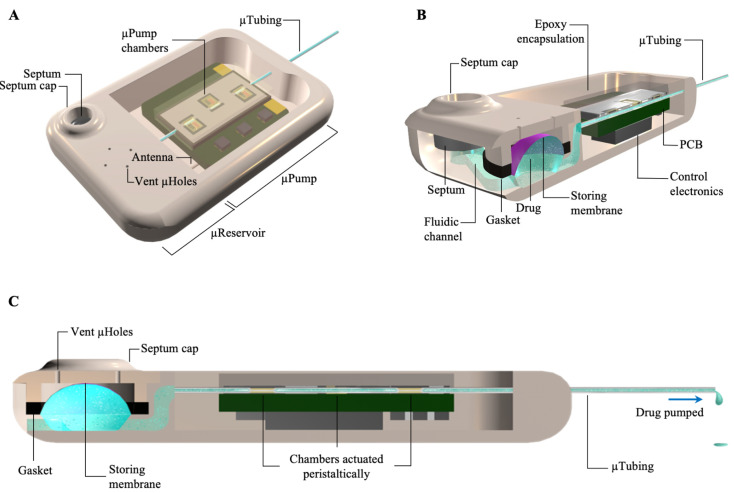
A schematic view of the pump microsystem. (**A**) An isometric view of the device showing the overall structure. (**B**) A cutaway view of the device showing the fluidic path from the refill port to the storing area and the micropump. (**C**) A side cutaway view of the device showing the fluidic path in the microcatheter from the reservoir, through the pumping chambers, and out to delivery.

The overall structure of the integrated device (micropump + microreservoir) was built using stereolithography (SLA) 3D-printing technology with a biocompatible resin and filleted exterior, optimized for long-term subcutaneous implantation. The structure of a 10 µL reservoir was embedded in the microsystem, while the micropump was fabricated separately and integrated into the device (Figure 1A). The microchannel between the refill port and the storing area is angled in a way that the inlet and outlet of the storing area are on opposing sides of the reservoir. This facilitates wetting the surfaces during filling/refilling and pushing the air pockets from the system (see Figure 1B). The inlet end of the catheter microtubing was glued to the outlet of the microreservoir and the outlet end of it was glued to the enclosure wall using biocompatible cyanoacrylate to ensure stress relief (see Figure 1C). A hole on the bottom side of the reservoir (not shown) was designed for placing the 5 mm antenna of the micropump.

To protect the system from the environment and to minimize the inflammatory or immune response, several considerations were applied. The structure was built with dental resin with a Young’s modulus of 2.7 GPa, providing enough mechanical strength for handling during the surgery. The space around the micropump and within the housing was filled with a biocompatible epoxy to ensure mechanical stability, a smooth top surface, and biocompatibility. Large fillets around the exterior of the device minimize mechanically induced inflammation following implantation when in contact with tissue. The top of the reservoir’s storing area is covered with a cap, protecting it from the mechanical stress and from inadvertent needle puncture during refill. Results of our stand-alone micropump characterization published recently demonstrated minimal impact of temperature on the micropump operation in the range of ±3 °C. Finally, the device was parylene-coated to protect the electronics from moisture and to enhance biocompatibility.

## 2. Results

### 2.1. Benchtop Experiments

**Figure 2:**
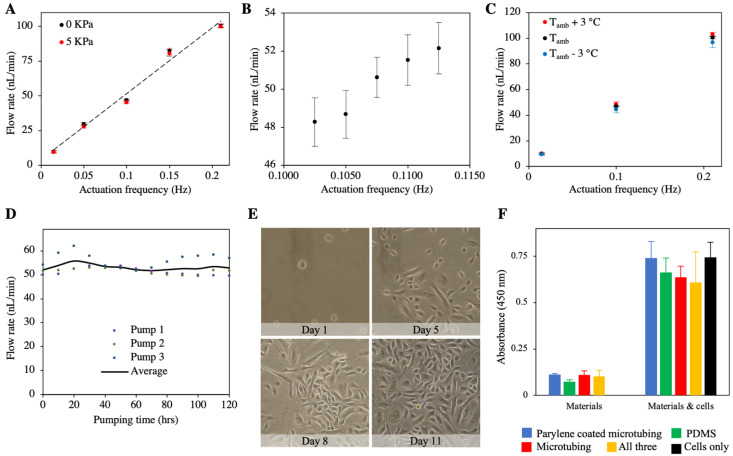
(**A**) Benchtop characterization of the micropump showed a linear (R^2^ = 0.98) relation between the actuation frequency and the flow rate, indicating effective control over the flow rate. The application of 5 kPa backpressure did not significantly change the flow rate. Error bars: standard deviation. (**B**) The micropump had a 2.35 nL/min resolution in delivering at the target flow rate of 50 nL/min. Error bars: standard deviation. (**C**) A ±3°C fluctuation in the ambient temperature had an insignificant impact on the flow rate. Error bars: standard deviation (**D**) The micropump could provide a consistent flow rate for 120 h after stabilization, with an average of 53.1 ± 1.8 nL/min (mean ± standard deviation), with fluctuations smaller than 5%. (**E**) In vivo biocompatibility experiments of the micropump components showed an increase in the numbers of proliferating cells and normal development of cell morphology over time. (**F**) Results of XTT assays with 120 min incubation time at Day 7 showed insignificant between-group differences for cell health, biocompatibility, and viability for any of the micropump component conditions, confirming biocompatibility of the micropump components. Error bars: standard error of the mean. (**A**–**C**,**E**,**F**) adapted from [36].

The micropump and the microreservoir were characterized separately using benchtop experiments presented previously [35,36]. The micropump characterization results showed a linear (R^2^ = 0.98) control over flow rate in the range of 10–100 nL/min by adjusting the actuation frequency, where applying backpressures as large as 5 kPa did not significantly change the flow rates (Figure 2A). To find the resolution of the drug delivery, the target flow rate of around 50 nL/min used in this study was tuned by changing the frequency, and it was found to be 2.35 nL/min (Figure 2B). Since the micropump works based on thermal phase-change peristalsis, the impact of a ±3 °C change in the ambient temperature was characterized, demonstrating an insignificant change in the flow rate ( <5% change) (Figure 2C). Further details on micropump characterization can be found in our previous article on the micropump technology [36]. The microreservoir characterization results demonstrated zero forward flow and insignificant backward flow (~2% of overall volume) due to membrane forces. The septum was shown to be capable of ~65,000 leak-free refill punctures under 100 kPa backpressure. Further information on the reservoir technology was reported in our microreservoir paper [35].

The micropump fabrication process was modified to improve functionality for long-term applications (details in Section 4.1). To explore the long-term functionality of the microsystem, the micropumps were tested in a simulated environment at the same temperature range and with analogous thermal properties to the murine subcutaneous implantation. The micropumps were set to run at 50 nL/min for 120 h, while the flow rates were tested every 10 h. Results of the tests on three micropumps showed a flow rate of 53.1 ± 1.8 nL/min (mean ± standard deviation) with fluctuations smaller than 5% (Figure 2D). It was observed that during the continuous operation, the set temperature of the micropump chambers had to be increased to maintain the target flow rate, until a stability point. On average, the micropumps required 23 h of continuous operation to stabilize.

The biocompatibility of the materials used in the micropump was studied and presented in our recent article on the micropump [36]. Briefly, in one experiment, the health and viability of the control cells were established. The growth of random samples of human mammary epithelial cells (ATCC-HMEC cell line) was recorded for 11 days. The results showed that the numbers of cells increased and cell morphology became more developed over time (Figure 2E). In another experiment, cell viability of the control cells (no micropump parts) versus the cells grown in the presence of key micropump components was performed using XTT assay using the ATCC-HEMC human epidermal epithelial cell line. The results of the XTT assay with 120 min incubation time for Day 7 are presented in Figure 2F. A comparison of the control bar (far right in the graph) and other bars indicated no statistically significant between-group differences for cell health, biocompatibility, and viability for any of the micropump component conditions. MTT and BRDU assays were performed and demonstrated similar results.

**Figure 3:**
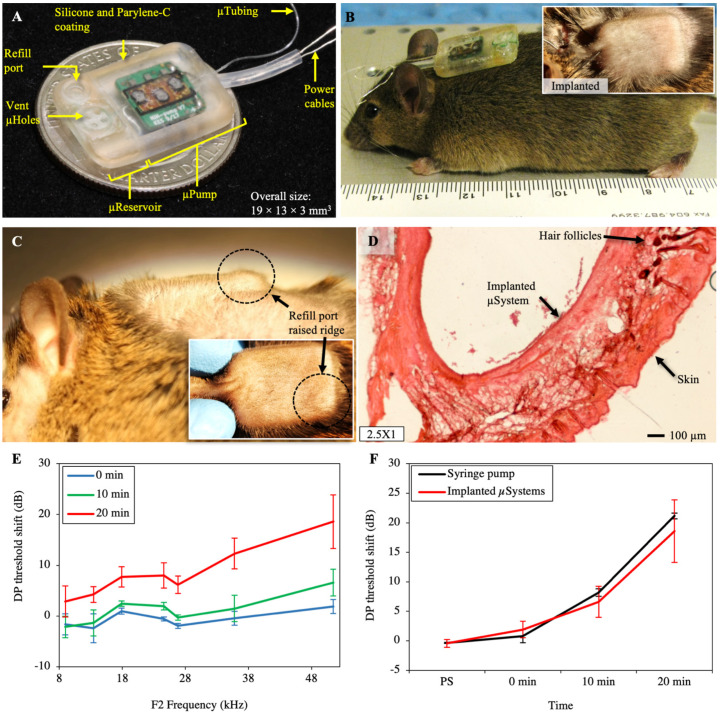
(**A**) Photograph of a completed microsystem with a 10 µL microreservoir and a micropump working in the range of 10–100 nL/min. This configuration of the microsystem was optimized for subcutaneous implantation in mice with an overall thickness of 3 mm. (**B**) Photograph of a microsystem before implantation demonstrating the width of the microsystem optimized for subcutaneous implantation in mice. Inset: An image of the microsystem after implantation via insertion of the microsystem in a small incision made in the center of the upper back of a mouse. (**C**) Side view and top view (inset) of a microsystem implanted showing the visibility of the refilling port. (**D**) A representative 60 µm section of the skin surrounding the microsystem showed no sign of any active inflammatory cells. A fibrotic layer (pink tissue) fully encapsulated the microsystem. (**E**) DPOAE threshold shifts (mean ± standard error of the mean) from four mice recorded from 0 to 20 min after the start of a salicylate infusion showed a systemic increase in DPOAE thresholds, demonstrating successful delivery of salicylate to the cochlear. (**F**) Results of the threshold shift (mean ± standard error of the mean) of the most basal region (F2 = 51.4 kHz) for implanted microsystems and the syringe pump showed perfect consistency, demonstrating the successful performance of the microsystem (*n* = 6 for syringe pumps, *n* = 4 for microsystems). PS: post surgery; 0 min: right after starting the pump; 10 and 20 min: 10 and 20 min after starting the infusion.

The completed integrated microsystem is shown in Figure 3A. The overall microsystem size measures 19 × 13 × 3 mm^3^ (L × W × H), enabling transdermal applications and subcutaneous implantation in small animal models such as mouse, due to its small size and planar form factor. The microsystems were set to run at 50 nL/min for 20 min in a glycerin bath at 30–34 °C then were turned off, and the backflow due to microreservoir membrane-restoring forces was measured following the methods described in [35]. The results showed an average flow rate of 54.3 nL/min, with backflows smaller than 2.5% of the microreservoir capacity, confirming the suitability of the device for in vivo experiments.

### 2.2. Implantation and Long-Term Biocompatibility

Figure 3B shows the implanted microsystem, demonstrating the small size and thickness of the device compared to a mouse. Figure 3C shows the raised ridge of the microsystem’s refill port, which is clearly distinguishable by slightly pulling the skin aside with two fingers. We also successfully refilled the reservoir while implanted to confirm transcutaneous access. The mouse recovered well from the surgery, and over the six-month survival period, was in good health. Postimplantation, the mouse was monitored daily, and the results indicated that the overall health was excellent. This included no signs of fever or infection, no significant swelling, no redness or lumps around the micropump location or incision, nor the presence of discolored fluid, and no weight loss. Cage behaviors, including feeding, grooming, and drinking, were normal. The mouse was photographed weekly during the six-month period.

The histological processing of the tissue surrounding the microsystem revealed a significant fibrotic layer around it, along with some ingrown hair follicles associated with the incision site. Otherwise, there were no cellular indications of infections or additional inflammatory responses, or abnormal cellular structures or responses. A typical section of the skin surrounding the microsystem is shown in Figure 3D. The skin surface is on the bottom, showing the normal dermal layers, with no sign of any active, inflammatory cells. The microsystem was in the cavity on the upper surface; here a fibrotic layer is seen (pink) that fully encapsulated the microsystem. This was the only abnormal feature of the tissue surrounding the microsystem after six months of survival, other than the dark hair follicles (upper right of the figure) associated with the incision site.

### 2.3. Inner-Ear Drug Delivery

Salicylate was delivered at the RWM of four mice with four different microsystems at 50 nL/min for 20 min while the distortion product otoacoustic emission (DPOAE) threshold shifts were recorded during the infusion. This replicated a previous study in our laboratory that was performed with a syringe pump [38]. A baseline measurement was acquired approximately 10–15 min before the cannula placement surgery, and all other DPOAE values reported here were relative to that baseline. Figure 3E shows the distortion product (DP) threshold shift at three time points: 0 min (the time at which the micropump was turned on), and 10 and 20 min following the onset of salicylate perfusion. A systemic shift in the DP threshold could be observed, which showed the successful delivery of salicylate to the cochlea. The results of the mean threshold shift of the most basal region (F2 = 51.4 kHz) at the same time points and the time point right after the surgery (PS: post surgery) for microsystems and the syringe pump are presented in Figure 3F. There were mean shifts of 6.6 and 18.6 dB after 10 min and 20 min infusion, respectively, for the implanted microsystems, consistent with the syringe pump results, suggesting successful performance of the implanted microsystem.

## 3. Discussion

The thin, miniature, and yet scalable microsystem presented here consisted of a wirelessly controlled programmable micropump and a refillable microreservoir for implantable murine inner-ear drug delivery. For surgery to be a minor procedure for an implantable device, the surface area of the device should be less than 10% of the animal’s overall surface area [39]. Our microsystem uses only 3.5% of the surface area of an average mouse with a 7000 mm^2^ surface area [40]. The overall weight of the microsystem is 1.58 g, which is less than 10% of the overall weight of an average-sized mouse (35 g) [41], an estimation that was used for implantable prototypes [42]. Further, our successful long-term implantation results confirmed that the device was thin enough (3 mm) for animal models as small as mouse. Compared to other devices used for inner-ear drug delivery (e.g., [6,43,44]), this microsystem reduced the overall size and thickness by factors of at least 4 and 2.7, respectively.

Although demonstrated for murine inner-ear drug delivery here, the novel design and fabrication method of this microsystem allows employing the concept for different applications in implantable and transdermal drug delivery ranging from mouse to humans. The enclosure and the microreservoir were built with a novel fabrication technique consisting of SLA 3D printing of a biocompatible resin, deposition of parylene-C on a biocompatible sacrificial layer, and micro-molding with SLA 3D-printed molds. SLA 3D printing enables the fabrication of features as small as 250 × 350 µm^2^, simply by creating the model in a computer-aided design (CAD) file and uploading it to the printer [45]. Since SLA 3D-printed components can be scaled by minimal modifications in the CAD file, and parylene deposition is a conformal process, this fabrication process can be simply scaled. In our previous work, we demonstrated reservoirs with capacities of 1, 10, and 100 µL and with only 3 mm thickness, and these can be fabricated in any size in between or larger sizes by adjusting the storing membrane thickness [35].

The micropump works based on the peristalsis concept and is built around a catheter microtubing directly on the backside of a printed circuit board (PCB), where the chambers are created using inkjet-printing technology and are actuated by thermally phase-changing material. All these features facilitate modularity and scalability, along with other benefits. The peristalsis eliminates the necessity of using valves, enabling a simple, modular, and scalable fabrication process by only creating chambers with different sizes along the microtubing for pumping. The chambers were created using inkjet-printing technology, in which the chamber is designed in 2D using CAD software and multiple layers of it are printed on the PCB. The chamber size and design can be scaled by simple modifications in the CAD design. Microtubing can be scaled while using the same fabrication process by choosing another tubing size (e.g., ID = 305 μm, OD = 635 μm; MRE025, Micro-Renathane^®^ Catheter Tubing, Braintree Scientific Inc., Braintree, MA, USA). The PCB and the amount of deposited wax can be simply scaled.

This drug-delivery concept can also be used for transdermal drug delivery by coupling the catheter microtubing to a needle and a needle injection mechanism, or by coupling the microtubing to an array of hollow microneedles at the bottom of the device or as a lateral extension. Successful functionality of the microreservoir for transdermal applications was shown in our previous publication [35]. The suitability of the microsystem for implantable applications regarding weight, overall size, and thickness shows it fits transdermal applications since transdermal devices do not need to meet such stringent standards. Further, the additive manufacturing technique utilized here enables creating a curved substrate to improve comfort when contacting the skin.

## 4. Materials and Methods

### 4.1. Fabrication Process

The overall structure of the device consisting of the microreservoir substrate adjacent to an open container for placement of the micropump was fabricated using SLA 3D printing (Form 2, Formlabs Inc., Somerville, MA, USA) with a biocompatible resin (Dental SG resin, Formlabs Inc., Somerville, MA, USA). To reduce the tissue irritation and improve long-term subcutaneous implantability, fillets of 1 mm radius for the external walls of the enclosure were designed for 3D printing, and during the postprocessing, the external surfaces were polished using sandpaper up to 2000 grit. This was followed by a 1 µm parylene-C deposition with a PDS 2010 LABCOATER™ 2 (Specialty Coating Systems, Indianapolis, IN, USA). The correlation between the parylene weight and the thickness of the deposited layers was evaluated using an optical profilometer (Nanovea ST400 3D Non-Contact Profilometer, NANOVEA, Microphotonics Inc., Allentown, PA, USA). It was found that at a 10 mTorr vacuum level, and 690, 175, and 135 °C set temperatures for the furnace, vaporizer, and chamber respectively, the thickness of the deposited layers was 1.2 µm per 1 g of parylene placed in the tool.

Polyethylene glycol (PEG, 1000 Mn, melting point: 33 °C, Sigma-Aldrich, St. Louis, MO, USA), a biocompatible and water-soluble material, was deposited in a dome shape as a sacrificial layer to define the microreservoir storage capacity of 10 µL. Polyurethane-based catheter microtubing (ID = 125 μm, OD = 250 μm; MRE010, Micro-Renathane^®^ Catheter Tubing, Braintree Scientific Inc., Braintree, MA, USA) was fixed to the outlet port of the microreservoir using biocompatible cyanoacrylate (2p-10, Fast cap, Ferndale, WA, USA). A second parylene-C coating with 5.6 µm thickness was deposited on top of the PEG sacrificial layer. A biocompatible silicone (MED-6215, NuSil™ Technology LLC, Carpinteria, CA, USA, 1:10 ratio) was used to micromold a gasket. The gasket was placed on the gasket ring around the edge of the storing area and was compressed with a 3D-printed storing cap, fixed to the structure using cyanoacrylate. The PEG sacrificial layer was molten and rinsed using hot deionized (DI) water, and the microtubing was gently removed. A biocompatible silicone (MED-6215, NuSil™ Technology LLC, Carpinteria, CA, USA, 1:10 ratio) was micromolded to create the septum with 1 mm thickness and 2.5 mm diameter, followed by parylene deposition. The septum was placed in the refill port and fixed with a 3D-printed septum cap. A compression ring was embedded in the septum cap to created lateral compression in the septum. For further details on the fabrication process, see [35].

The mechanical structure of the micropump was direct-write printed on the back of a 0.7 mm standard PCB. Standard PCB assembly technology was utilized to populate the control electronics on the front side of the PCB. Three pairs of resistors and thermistors were assembled on the back of the board in a linear offset formation to provide subsequent placement of the microtubing. The micropump structure was 3D-printed using a polymer inkjet printer (Roland VersaUV LEF-12, Tokyo, Japan) with an ECO-UV^®^ resin, creating three chambers with 1.14 mm diameter and 0.35 mm height around the resistor/thermistor pairs, along with a groove for the microtubing. This printing technology enables placing the substrate (i.e., the backside of the PCB) on the printer platform and simply printing tens of layers on top of it to make the 3D structure. The SLA 3D-printing technology, on the other hand, prints the structure directly on the device build platform, precluding printing on a custom substrate due to complications regarding attaching the substrate to the build platform, alignment, height offset, and submersion of the substrate in the liquid resin, which damages substrates that involve electronics. We evaluated the height of the printed structure using this printer, by printing multiple layers of the same structure over blank PCB. We found the average height to be 7.7 µm per layer, necessitating the printing of 45 layers to achieve a 0.35 mm height for the chambers.

Polyurethane-based catheter microtubing (ID = 125 μm, OD = 250 μm; Micro-Renathane Catheter Tubing, Braintree Scientific Inc., Braintree, MA, USA) was placed, fixed, and sealed in the groove using the biocompatible epoxy resin (EPO-TEK 301-2, Epoxy Technology, Boston, MA, USA). To achieve precision in wax deposition, docosane (C_22_H_46_, Sigma-Aldrich, St. Louis, MO, USA) was micromolded in a Tygon tubing (1.52 mm O.D and 0.51 mm ID). For each chamber, 6 mm of the wax molding tubing was cut (wax volume: 1.2 µL) using a sharp razor blade, and the wax was gently pushed out using a blunt needle tip. The micropump was placed on a heater at 70 °C and the wax in the chamber, followed by placing the pump in a heated desiccator for 10 min for debubbling of the wax. The debubbled molten wax was solidified slowly, at a rate of 0.6 °C/min using a temperature controller developed in our laboratory. A thin (viscosity: 10–35 cP) visible light-cure biocompatible adhesive (Loctite 4306, Rocky Hill, CT, USA) was placed on the wax and desiccated for 5 min to fill any voids in the wax, and cured slowly by exposure to fluorescent light in the laboratory for 1.5 h. To enhance the mechanical strength of the chambers, the top of the chambers was covered with biocompatible epoxy resin (EPO-TEK 301-2, Epoxy Technology, Boston, MA, USA), debubbled for 2 min, and cured at 30 °C for 24 h. The micropump was encapsulated with an approximately 2 μm layer of parylene-C (Specialty Coating Systems, Indianapolis, IN, USA). The parylene-C coating provided a moisture barrier for testing the micropumps before integration.

The micropump was carefully placed in the designated container in the enclosure and fixed with biocompatible cyanoacrylate (2p-10, FastCap, Ferndale, WA, USA). The inlet end of the microtubing was placed, fixed, and sealed to the microreservoir outlet using biocompatible cyanoacrylate (2p-10, FastCap, Ferndale, WA, USA). An oval-shaped hole was created in the outlet end of the microsystem for the power cables of the micropump. A biocompatible silicone tubing (0.762 mm ID, 1.65 mm OD, Bio-Sil 1450 Silicone Tubing, Saint Gobain, MA, USA) was used to encapsulate the power cables to create a smooth soft surface in contact with the tissue. The power cables were passed through this 1 cm long silicone tubing and fixed and sealed with biocompatible cyanoacrylate (2p-10, FastCap, Ferndale, WA, USA). The micropump container in the enclosure was washed thoroughly with isopropyl alcohol, dried, and filled with biocompatible epoxy resin (EPO-TEK 301-2, Epoxy Technology, Boston, MA, USA) and cured at 30 °C for 24 h. The integrated device was encapsulated with a 1 μm layer of parylene-C to provide a biocompatible moisture barrier around the device.

### 4.2. Benchtop Experimental Methods

The micropump and the microreservoir were independently characterized using benchtop experiments, which are briefly presented in the Results section. We also presented benchtop testing of the improved fully integrated micropump and reservoir for a murine inner-ear drug delivery paradigm. This testing followed an in vivo protocol originally developed and demonstrated with a syringe pump delivering a reversible ototoxic drug, sodium salicylate, to the RWM niche of mice at a rate of 50 nL/min for 20 min (1 µL total volume) [38].

To simulate the in vivo environment, a temperature controller was built (details in Appendix A) to control the temperature of different liquid models. To find the temperature variations and thermal heat loss of the subcutaneous environment, experiments with a dummy pump were performed in vivo and were repeated in vitro, and the results were compared (details in Appendix B). The micropumps were tested for their long-term performance, during which the on/off temperatures of the chambers and the actuation frequency were set for delivery of 50 nL/min. The micropumps were placed in a custom-made SLA 3D-printed fixture in a Petri dish, and submerged in glycerol with a set temperature of 32 °C provided by the custom-made temperature controller. The testing was performed following the protocol previously developed in our laboratory and reported in [36].

The microsystem was tested to confirm its suitability for in vivo experiments. A vacuum-assisting filling method optimized for this device was used to fill/refill the microreservoir. The downstream of the microtubing was connected to an empty 3 mL syringe. The plunger was pulled away to generate a vacuum in the system. A 30 gauge sharp needle connected to a 100 µL syringe (1710 RNR SYR, Hamilton Co., Reno, NV, USA) was used to pierce through the septum and allow the vacuum to suck the fluid from the syringe and remove bubbles in the system. The downstream pressure was set back to atmospheric pressure by disconnecting the 3 mL syringe, and the fluid was injected into the system by gently pushing the syringe plunger until the cavity membrane of the microreservoir inflated to full capacity, confirmed by observing fluid leaving the device via the microtubing. The needle was removed from the septum. The microsystems were turned on to pump at 50 nL/min, and after 20 min of operation it was turned off and the backflow in the microreservoir was found by observing the fluid downstream of the microtubing.

### 4.3. In Vivo Experimental Methods

The microsystem was tested *in vivo* to show its suitability for subcutaneous long-term implantation in mice, and functionality for acute drug delivery to the mouse inner ear. To evaluate long-term biocompatibility for subcutaneous implantation, the microsystem was implanted in a mouse model for six months, and frozen sections of surrounding tissue were analyzed histologically for signs of inflammation. The drug-delivery test was performed following a protocol developed by our laboratory [38] for the administration of sodium salicylate to the RWM, leading to temporary hearing loss—reversible shifts of otoacoustic emission thresholds and levels. Salicylate can act as a competitive antagonist at the anion-binding site of prestin [46], which causes reversible disruption of outer hair cell motility, resulting in a 20–30 dB sensorineural hearing loss. The disruption of prestin results in a reduction in DPOAE amplitudes and thresholds. DPOAEs are a functional readout of outer hair cell viability.

#### 4.3.1. Subcutaneous Implantation and Biocompatibility

Mice were anesthetized with ketamine (Covetrus, Portland, ME, USA) and xylazine (Akron, Lake Forest, IL, USA), 120 and 10 mg/kg body weight, respectively, in combination with topical application of 4% lidocaine (Akron, Lake Forest, IL, USA) for analgesia for the implant surgery. The microsystem was rinsed with sterilized double-distilled water and air-dried, followed by exposure to ultraviolet light for 2 h (1 h each side) using a certified biosafety cabinet (Thermo Fisher Scientific, Thermo Scientific™, model: 1300 Series Class II, Type A2, Marietta, OH, USA). The suitability of this sterilization method was confirmed in our previous publication on the micropump, in which the individual components of the device were sterilized, and biocompatibility studies were successfully performed on them [36]. 

Mice were positioned on a servo-controlled heating pad maintaining aseptic conditions, and when the proper plane of anesthetic was achieved (toe pinch, heart rate, respiratory rate), the upper back was shaved and cleaned, and the microsystem was inserted subcutaneously in the center of the upper back via a small incision. Supplementary doses at one-third of the initial dose were administered as needed to maintain the proper levels of general anesthesia. Following insertion ventral to the dermis, medical-grade adhesive (Loctite 4206, Rocky Hill, CT, USA) was used to secure the wound closure, along with several stitches to close the incision over the microsystem. The mouse was returned to a single house cage and monitored every day for the first two weeks and then weekly for 6 months. The surgical site was photographed, mobility was observed, and weight was measured. After six months, the mouse was euthanatized (0.22 ml/kg, IP, Euthasol^®^, Covetrus, Portland, ME, USA) and then perfused with 4% paraformaldehyde fixative (Thermo Fisher Scientific, Thermo Scientific™, Marietta, OH, USA) transcardially. After six months of implantation, tissue samples were dissected from around the microsystem and prepared for standard frozen sectioning followed by hematoxylin + eosin (H&E) tissue staining to visualize cellular locations and structure around the microsystem. All animal procedures were approved by the University of South Florida Institutional Animal Care and Use Committee and were performed using the National Institutes of Health and veterinary standards of care.

#### 4.3.2. Inner-Ear Drug Delivery

Four young adult (2–4 months of age) CBA/CaJ mice bred and raised in-house were used for this study. The salicylate solution consisted of NaCl (120 mM), KCl (3.5 mM), CaCl_2_ (1.5 mM), glucose (5.5 mM), HEPES buffer (4-(2-hydroxyethyl)- 1-piperazineethanesulfonic acid, 20 mM), and sodium salicylate (50 mM). The pH was adjusted to 7.5 using NaOH. All solutions were prepared on the day of the experiment using sterile double-distilled water. The salicylate was loaded into a 25 µL sterilized syringe (1702 LT SYR, Hamilton Co., Reno, NV, USA) with a needle (33 Ga, 7747-01, point style 4, 12°, 1″, Hamilton Co., Reno, NV, USA) and was debubbled. The microsystems were sterilized following the method described in Section 4.3.1. To fill the microreservoir, the vacuum-assisted filling method was used, with the fluid set at 1 mm from the microtubing tip.

Microsystems were subcutaneously implanted in the center of the upper back. A bullaostomy surgery was performed to prepare a site for infusion of the salicylate into the middle ear cavity. A mixture of ketamine (120 mg/kg body weight, Covetrus, Portland, ME, USA) and xylazine (10 mg/kg body weight, Akron, Lake Forest, IL, USA) injected via the intraperitoneal route to deeply anesthetize the mice for the bullaostomy surgery. The left ventral surface of the neck was then shaved and cleaned. Surgery was performed on the left (ipsilateral) ear following procedures described by Borkholder et al. [38]. During infusions, each mouse was anesthetized with supplementary doses at one-third of the initial dose administered as needed to maintain the proper levels of general anesthesia.

The microsystem was powered by a 3.7 V rechargeable Li-ion battery and was connected to a custom-made Android application using Bluetooth wireless communication. The actuation sequence and set temperatures were confirmed previously in benchtop experiments for a 50 nL/min infusion rate. After monitoring the microsystem temperature using the Android application for a stable temperature, the micropumps were activated to start the infusion. Auditory function was assessed via automated DPOAE threshold measurements using F2 frequencies of 8.9 kHz, 13.5 kHz, 17.9 kHz, 24.6 kHz, 35.8 kHz, and 51.4 kHz. Baseline DPOAEs were acquired before the surgery to compare to subsequent DPOAE threshold shifts following microsystem implantation. Further details of auditory function assessment were presented in our previous work [36,47].

## 5. Conclusions

We developed a microsystem for implantable and transdermal drug delivery with wireless control using 3D-printing technology. The micropump employed the peristalsis concept, with three chambers actuating sequentially using a phase-change actuation mechanism, built around a catheter microtubing to provide a leak-free microscale fluidic path. The micropump structure was created on the backside of a PCB that drove and controlled actuation and provided wireless control. The microreservoir was fabricated using 3D-printing technology with a thin dome-shaped parylene-C membrane with a minimal backflow due to fluidic discharge, which enabled integration to normally open pumping mechanisms, such as our micropump. The two components were integrated using 3D-printing technology in a miniature and thin form factor. In one configuration, the microsystem was optimized for implantation in a mouse animal model for inner-ear drug delivery, with a micropump with flow rates ranging from 10 to 100 nL/min and a microreservoir with a 10 µL capacity. In vitro experiments confirmed the suitability of the microsystem for murine inner-ear drug-delivery application. In vivo long-term murine implantation results showed no significant inflammation or infection after six months of implantation. An in vivo experiment was performed by subcutaneous implantation of the microsystem in mice with the catheter microtubing of four different microsystems in the RWM of four mice for delivery of sodium salicylate. The results indicated that the hearing (DPOAE response) was consistent across different micropumps and different animals, and results were consistent with syringe pump experiments performed with the same protocol previously performed in our laboratory.

The in vivo success of our novel and advanced microsystem for murine inner-ear drug delivery indicated translational potentials for the use of this technology in larger mammals, and clinically. 3D-printing technology as the key fabrication method here enabled building an extremely miniature and thin microsystem for miniature applications that were shown in this work as examples, while providing scalability for other applications in various animal models and clinical applications. The microreservoir structure was shown to be scalable in the range of 1–100 µL [35], while maintaining key performance criteria. The micropump is inherently scalable by appropriate scaling of the catheter microtubing size and actuation chamber volume. Future work will focus on long-term implantation of the microsystem for murine inner-ear drug delivery with periodic delivery of otoprotective compounds.

## Figures and Tables

**Figure A1:**
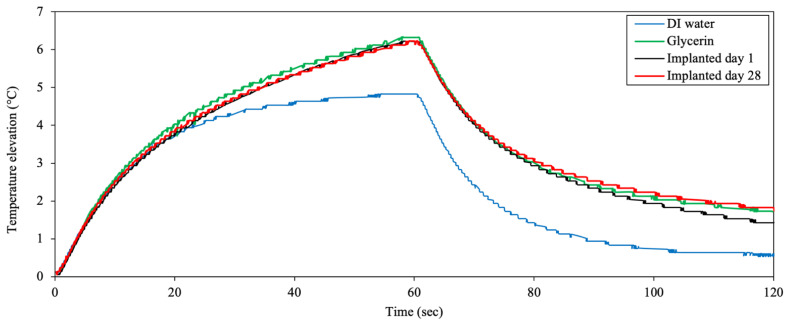
Temperature elevation in one of the side chambers due to heating of the middle chamber in different environments was studied to evaluate changes in the thermal properties of the subcutaneous environment over time and to find proper material for simulation of this environment for benchtop experiments. Comparison between the results of Day 1 and Day 28 after subcutaneous implantation showed insignificant changes in the thermal properties of this environment over time. Comparison of the results of the implanted data and the microsystem submerged in water and glycerin demonstrated suitability of glycerin for simulation of the thermal properties of the implanted environment for benchtop experiments.

## Data Availability

Data is contained within the article.

## Appendix A. Temperature Controller

To simulate the environment of the subcutaneous implant from a thermal perspective, a custom-made temperature controller was developed in our lab, enabling the submerging of the microsystems in a liquid environment with ~60 mL volume and at an adjustable set temperature with less than 0.2 °C temperature variation. The precise temperature control within ±0.1 °C was achieved by implementing a proportional-integral-derivative (PID) control using an Atmega328 microcontroller on a Peltier module along with a heat sink assembly and fan. For the temperature measurement, a commercially available temperature sensor (Surface Temperature sensor, STS-BTA, Vernier Software & Technology, Beaverton, OR, USA) based on a negative temperature coefficient thermistor with a resolution of 0.03 °C (at 0 to 40 °C) was used for the temperature measurement as an input to the PID control. The setpoint of the PID was entered by the user with an LCD-based user interface. The output computed by the PID algorithm provided a pulse-width modulation signal in conjunction with an H-bridge, to drive the Peltier and perform heating or cooling. A PCB was fabricated to execute the H-bridge using the N-channel and P-channel MOSFETs as switches. The PID was tuned and optimized by setting the ideal PID tuning parameters: proportional gain, integral gain, and derivative gain to achieve an overshoot of less than 3 °C; and a steady-state temperature variation less than 0.2 °C. The device was characterized for the slow reduction in temperature (0.6 °C/min) for pump fabrication and long-term temperature stability in the range of 16 °C to 37 °C for pump fabrication and testing.

## Appendix B. Thermal Simulation of Subcutaneous Environment

To find the temperature variations and thermal heat loss of the subcutaneous environment, experiments were performed in vivo and were repeated in vitro, and the results were compared. A dummy microsystem was fabricated with chambers filled with epoxy and no reservoir or microtubing. The microsystem was subcutaneously implanted, and an experiment was performed in which the middle chamber was activated for 1 min and then turned off. The changes in temperatures of the three chambers were recorded to be compared with the in vitro results. The temperature elevation of the side chambers was considered as the criterion for comparison of different cases. This experiment was performed over a period of a four weeks, and the tests were performed weekly. Then the microsystem was tested in vitro, during which it was submerged in DI water and glycerin, and the temperature and thermal properties were monitored by repeating the same experiments. Results showed that the thermal properties in the subcutaneous environment did not change significantly (<2%) over a period of four weeks. In addition, a comparison of the benchtop and in vivo results showed that glycerin and DI water could simulate murine subcutaneous in vivo environment with ~2% and 20% error, respectively, indicating the suitability of glycerin (see Figure A1).
